# Probiotic Dairy Dessert With Stevia and Sage Mucilage

**DOI:** 10.1002/fsn3.70986

**Published:** 2025-09-21

**Authors:** Mohammad Mahdi Asadi Garmaroudi, Marjaneh Sedaghati, Mohamad Javad Shakouri

**Affiliations:** ^1^ Department of Food Science and Technology NT.C., Islamic Azad University Tehran Iran

**Keywords:** dairy dessert, *probiotic*, sage seed mucilage, stevia

## Abstract

This study investigated the effects of different stevia‐to‐sugar ratios (SSR: 0, 0.001, and 0.006 *w/w*) and sage seed mucilage (SSM: 0%, 0.15%, and 0.3% *w/w*) on the physicochemical, microbial, and sensory properties of a probiotic dairy dessert during storage periods of 1, 10, and 20 days. Parameters assessed included pH, dry matter, fat, protein, total sugar, caloric content, phase separation, viscosity, color, total phenolic content (TPC), antioxidant activity (AO), probiotic viability, and sensory characteristics. Replacing sugar with stevia significantly reduced sugar content and calories, enhancing the health profile of the dessert. However, higher stevia levels (SSR 0.006) adversely affected texture by decreasing viscosity and increasing syneresis. In contrast, increasing the concentration of SSM improved viscosity and reduced syneresis, contributing to better texture stability. Both stevia and sage seed mucilage increased total phenolic content and antioxidant activity, thus adding nutritional value. The viability of *Lactobacillus casei* ( *L. casei* ) was maintained at the lower SSR (0.001), while the higher stevia substitution (0.006) reduced probiotic survival. Higher SSM concentrations positively influenced probiotic viability over storage, although a gradual decline was observed in all samples. Sensory evaluation indicated that the sample containing 0.001 SSR and 0.3% SSM on day 10 achieved the most favorable balance of taste, texture, and overall acceptability, closely resembling the control. These findings demonstrate that moderate sugar substitution with stevia and the addition of sage seed mucilage can enhance both the functional and sensory qualities of probiotic dairy desserts, promoting consumer acceptance and healthier product development.

## Graphical abstract

This study evaluated a probiotic dairy dessert with varying stevia‐to‐sugar ratios and sage seed mucilage levels during storage. Moderate stevia substitution reduced sugar and calories while maintaining probiotic viability and sensory acceptability. Sage seed mucilage enhanced texture stability by improving viscosity and reducing syneresis, and both ingredients increased antioxidant activity and phenolic content, boosting the dessert's nutritional and functional value.

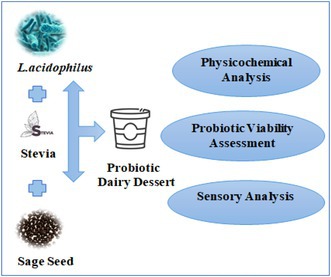

## 1 Introduction

In recent years, there has been a significant increase in consumer awareness regarding the relationship between diet and health. Consequently, producing food with health benefits has become essential, and individuals are actively following to consume such foods in their diets to sustain or enhance their quality of life. The concept of probiotic foods encompasses foods or additives that not only provide nutritional value but also offer health benefits to the consumer, such as lowering the risk of chronic disease development. The health‐promoting effects of probiotic foods are primarily due to their content of beneficial microorganisms, which exert specific biological activities that positively influence human physiology (Kumar et al. 2023; Aziz et al. 2024; Amin et al. 2025).

Dairy desserts, which are typically composed of at least 50% fresh cow's milk or reconstituted and recombined milk, are formulated with thickeners (such as starch and hydrocolloids), sugar, flavorings, and colorants, and are subjected to heat treatment. These desserts contribute significantly to a healthy diet and overall well‐being, as they are rich sources of essential nutrients, including calcium, vitamin D, phosphorus, potassium, manganese, riboflavin, and niacin. Due to their nutritional benefits and appealing taste, dairy desserts are popular among diverse consumer groups and help add variety to daily food choices. Additionally, dairy desserts can serve as an excellent option for producing probiotic products, providing a delicious and convenient vehicle for delivering beneficial microorganisms to consumers (Hosseiny and Sedaghati 2023).

Stevia is a calorie‐free sweetener with a glycemic index of zero, commonly used to sweeten dairy products like yogurt, milk‐based desserts, and ice cream. The main components of stevia, which comprise stevioside, rebaudioside A, rebaudioside C, and dulcoside A, are 200–300 times sweeter than regular sugar (sucrose). Stevia is stable under heat, withstanding temperatures up to 200°C (392°F), making it suitable for use in heat‐processed foods. Additionally, stevia supports weight management, is safe for individuals with diabetes, and is not absorbed in the small intestine during digestion (Kotebagilu et al. 2022). Wild sage seed (*S. macrosiphon*) is a small, round seed that quickly absorbs water, swells, and forms a mucilaginous layer. The mucilage extracted from sage seed (SSM) is particularly valued for its high hydrocolloid content, which contributes to its exceptional thickening and gelling abilities. These natural hydrocolloids are increasingly incorporated into innovative food products, where they help improve texture and stability, meeting the evolving preferences of today's consumers (Abbasi Saadi et al. 2024).

Limited research has used various hydrocolloids as stabilizers and stevia as a sweetener to produce low‐calorie desserts. Studies have investigated the replacement of sugar with stevia in Indian desserts (Kotebagilu et al. 2022) and the incorporation of inulin and stevia in low‐calorie chocolate dairy desserts (Seyed Mahmoodzade and Ahmadi 2021). Additionally, the effects of stevia–sucralose and inulin on the physicochemical properties of sugar‐free and low‐fat dairy desserts have been examined (Rodriguez Furlán and Campderrós 2017). The present study produced probiotic dairy dessert with *Lactobacillus casei* ( *L. casei* ), different stevia (0%, 0.01%, and 0.03%) and SSM (0%, 0.15%, and 0.3%) conditions. Also, the viability of the probiotic bacteria, as well as the physicochemical, microbial, and sensory properties of the probiotic dairy dessert, were assessed during storage.

## 2 Materials and Methods

### 2.1 Material

Fresh and chilled bovine milk with 1% fat content, along with milk powder, was purchased from Pegah Company. Food‐grade stevia powder was supplied by Sinochem Company in China, while sage seeds were sourced from Nature Green Cycle Company in Bushehr, Iran. Food‐grade gelatin and sugar were obtained from Golha Company. Cultures of *Lactobacillus casei* PTCC 1608 were acquired from the Iranian Research Organization for Science and Technology (IROST) in Tehran. Lyophilized *L. casei* (exceeding 10^8^ CFU/mL) was cultured in MRS medium for 24 h at 37°C in a CO_2_ incubator (Memmert, Munich, Germany). MRS agar and broth (Man, Rogosa, and Sharpe medium) were provided by Merck (Darmstadt, Germany). All chemicals used in this study were of analytical grade and sourced from Merck (Germany).

### 2.2 Preparation of **SSM**

To extract SSM, the seeds were combined with distilled water at a ratio of 1:59 (seed: water, *w/w*) and incubated in a water bath at 25°C overnight. The resulting mixture was filtered through a 40‐mesh stainless steel sieve to remove solid residues. The filtrate was then mixed with ethanol at a ratio of 10:30 (filtrate: ethanol, *v/v*) and allowed to stand for 30 min to facilitate gel formation. The mucilage gel was subsequently separated by centrifugation (Funke Gerber, Germany) at 3000 rpm for 20 min and dried using a laboratory oven (Memmert, Germany) at 70°C for 24 h (Bayat et al. 2022).

### 2.3 Probiotic Milk Dessert Production

To prepare the milk dessert, low‐fat milk (75 g) was combined with varying amounts of stevia (0, 0.01, and 0.03 g) and sugar (10, 8.3, and 5 g), corresponding to SSR of 0, 0.001, and 0.006 (w/w), respectively. Gelatin (2 g), milk powder (12 g), and SSM at concentrations of 0%, 0.15%, and 0.3% (*w/w*) were also added slowly to the milk mixture. The mixture was thoroughly stirred using a magnetic stirrer (IKA, Staufen, Germany) at 250 rpm to ensure uniform dispersion and heated at 85°C for 10 min. After heating, the samples were cooled to 40°C, and a *L. casei* inoculum (10^8^cfu/mL) was added. The mixtures were then cooled to 15°C under continuous stirring, transferred into sterile containers, sealed, and stored at refrigeration temperature for 20 days. Changes in some measured parameters were evaluated on days 1, 10, and 20 (Hosseiny and Sedaghati 2023).

### 2.4 Physicochemical Analysis of Probiotic Milk Dessert

Probiotic milk dessert samples were examined throughout the storage period for pH, dry matter, ash, fat, protein, total sugar, and calorie content. Dry matter was measured using the oven‐drying technique (Memmert UN 110, Germany). Fat and protein levels were assessed by the Gerber and Kjeldahl methods, respectively, while total sugar was determined using the Fehling method. All analyses were performed in triplicate to ensure accuracy (Schiatti‐Sis'o et al. 2023).

Syneresis was measured by centrifuging 10 g of the sample and calculating the percentage of the supernatant liquid obtained after spinning the sample for 20 min at 2790 g. The apparent viscosity of the milk desserts was evaluated using a viscometer (DV II + LV, Brookfield, Middleboro, MA, USA) fitted with an LV4 spindle. Samples were placed in the measuring container, and viscosity readings were taken at 14 different spindle speeds, ranging 1.5–100.0 rpm, all conducted at 20°C (Hosseiny and Sedaghati 2023). All tests were performed in triplicate.

### 2.5 Color Assessment of Probiotic Milk Dessert

To assess the color attributes of probiotic milk dessert, the Hunterlab device (UltraScanvis, US‐Vis 1310, USA) was utilized to measure the L*, a*, and b* values. The lightness (L*) scale ranged from 0 (black) to 100 (white), a* value spanned from +127 (red) to −128 (green), and the b* value extended from +127 (yellow) to −128 (blue) (Zonoubi and Goli 2020).

### 2.6 Total Phenolic Content and Antioxidant Activity

Milk dessert samples were centrifuged and filtered before measuring total phenolic content (TPC) using the Folin–Ciocalteu method, with results expressed as gallic acid equivalents per liter. Antioxidant activity (AO) was assessed by mixing the samples with DPPH solution, measuring absorbance at 517 nm, and calculating the percentage of inhibition. The IC_50_ value, indicating the concentration required to inhibit 50% of DPPH radicals, was determined from a standard curve based on inhibition percentages at different sample concentrations (Papaefthimiou et al. 2023).

### 2.7 Probiotic Bacteria Viability Assessment

For microbiological analysis, 10 mL of the mixed samples was homogenized in a sterile beaker containing 90 mL of sterilized saline solution (0.95% w/v) to create the initial 10^−1^ dilution. Subsequent serial decimal dilutions were prepared using the same saline solution. To enumerate *L. casei* , these dilutions were plated using the pour plate method on MRS agar supplemented with vancomycin (10 mg L^−1^). The plates were then incubated in a CO_2_ incubator at 37°C for 72 h. Results were reported as Log CFU per gram (Arabshahi and Sedaghati 2022).

### 2.8 Sensory Analysis

A panel of 12 trained evaluators, consisting of six males and six females aged 20–30 years, performed a sensory assessment using a 5‐point hedonic scale, where 1 indicated “extremely dislike” and 5 represented “extremely like.” The sensory attributes evaluated included color, taste, texture, and overall acceptability. These evaluations were conducted over a 20‐day storage period. For testing, 20 g of dessert samples were placed on labeled plates and stored at 4°C ± 1°C before being presented to the panelists alongside their meals. After each tasting session, panelists cleansed their palates by rinsing with water (Hosseiny and Sedaghati 2023).

### 2.9 Statistical Analysis

All experiments were conducted using a fully randomized three‐factor factorial design to evaluate the individual and interactive effects of stevia concentration, SSM concentration, and storage time on the physicochemical, microbial, and sensory properties of the probiotic dairy dessert. Data were analyzed using statistical software (SPSS, version 22, 2016). Significant differences were identified using Duncan's multiple range test at a 95% confidence level (*p < 0.05*).

## 3 Results and Discussion

### 3.1 Physicochemical Composition of Dairy Dessert

Table 1 presents the physicochemical properties of milk dessert samples formulated with varying concentrations of stevia and sage seed mucilage (SSM). The pH values of all samples ranged 6.7–6.78, which aligns well with the acceptable pH range for dairy desserts (6.3–6.8) as specified by the Iranian National Standardization Organization (INSO) (ISIRI 14681, 2012). A significant decrease in pH was observed with increasing concentrations of stevia (*p < 0.05*). Although the addition of SSM caused a decrease in pH, this reduction was not statistically significant, likely due to the low concentration used in this study. This finding is consistent with previous research indicating that stevia substitution lowers the pH in dairy products, while SSM may promote the growth of lactic acid bacteria (LAB), stimulating organic acid production during fermentation (Denina et al. 2014; Nasiri et al. 2021).

**TABLE 1:** The effect of stevia/sugar ratios (SSR) and sage seed mucilage on physicochemical properties of probiotic dairy dessert.

Samples	pH	Dry matter	Fat (%)	Protein (%)	Total sugar (%)	Calorie (cal)
Control	6.78 ± 0.05^a^	39.81 ± 0.2^a^	1.06 ± 0.3^a^	4.90 ± 0.8^a^	35.08 ± 0.5^a^	434.2 ± 0.05^a^
S_0_M_1_	6.78 ± 0.07^a^	39.93 ± 0.5^a^	1.07 ± 0.1^a^	4.91 ± 0.1^a^	35.31 ± 0.1^a^	434.5 ± 5^a^
S_0_M_2_	6.76 ± 0.02^a^	40.05 ± 0.3^a^	1.07 ± 0.2^a^	4.90 ± 0.2^a^	35.96 ± 0.5^ab^	434.9 ± 5^a^
S_1_M_0_	6.74 ± 0.04^b^	37.64 ± 0.2^b^	1.07 ± 0.6^a^	4.90 ± 0.6^a^	33.39 ± 0.5^b^	431.3 ± 5^ab^
S_1_M_1_	6.74 ± 0.05^b^	37.62 ± 0.5^b^	1.07 ± 0.4^a^	4.92 ± 0.6^a^	33.84 ± 0.4^b^	431.6 ± 4^ab^
S_1_M_2_	6.73 ± 0.04^b^	37.73 ± 0.6^b^	1.07 ± 0.4^a^	4.90 ± 0.5^a^	33.91 ± 0.6^bc^	431.8 ± 6^ab^
S_2_M_0_	6.72 ± 0.02^c^	34.20 ± 0.5^c^	1.07 ± 0.4^a^	4.90 ± 0.5^a^	31.15 ± 0.4^c^	428.1 ± 5^b^
S_2_M_1_	6.71 ± 0.04^c^	34.74 ± 0.3^c^	1.08 ± 0.4^a^	4.91 ± 0.8^a^	31.42 ± 0.2^c^	427.8 ± 5^b^
S_2_M_2_	6.70 ± 0.05^c^	34.93 ± 0.1^c^	1.08 ± 0.2^a^	4.92 ± 0.7^a^	31.89 ± 0.5^cd^	427.9 ± 5^b^

*Note:* “S” refers to stevia/sugar ratios (0, 0.001, and 0.006) and “M” refers to sage seed mucilage content (0%, 0.15%, and 0.3%).

^a–d^ Means within each column followed by different letters (A–D) show significant differences (*p* < 0.05) among treatments.

The dry matter content of the desserts increased significantly with higher levels of sugar replacement by stevia (*p < 0.05*), whereas variations in SSM concentration had no significant effect on dry matter (*p > 0.05*). This can be explained by the fact that sugar, a pure carbohydrate, contributes substantially to the total solids and dry matter in dairy desserts. Stevia, being a high‐intensity sweetener used in much smaller quantities and containing fewer solid components, reduces the overall solids content when substituted for sugar, thereby lowering dry matter (Schiatti‐Sisó et al., 2023). Additionally, the effect of Jujube mucilage on the increased dry matter and water holding capacity of yogurt has been previously reported (Yekta and Ansari 2019).

Neither increasing the proportion of stevia nor varying SSM levels significantly affected the fat and protein contents of the milk desserts, indicating that these macronutrients remain stable despite sweetener and hydrocolloid modifications. Regarding this, research reported an insignificant reduction in yogurt fat content with higher Jujube mucilage levels, whereas the protein content decreased significantly (Yekta and Ansari 2019).

Regarding sugar content and caloric value, increasing the proportion of stevia significantly reduced both total sugar content and calories (*p < 0.05*). This is expected since stevia contains little to no carbohydrates, and thus replacing sugar with stevia lowers the dessert's sugar and caloric content (Schiatti‐Sisó et al., 2023). Conversely, higher SSM levels significantly increased the measured total sugar content (*p < 0.05*), although its effect on caloric value was not significant. This is because SSM is primarily composed of polysaccharides and soluble fibers, which contribute to total sugar measurements but are largely indigestible dietary fibers, resulting in minimal caloric contribution (Bostan et al. 2010; Renall et al. 2024). Recent studies have also demonstrated the successful production of low‐calorie ice cream by substituting sugar with stevia, highlighting the potential of stevia as a sugar alternative in dairy desserts (Shenana 2022).

### 3.2 Change in Syneresis and Viscosity of Dairy Dessert

**FIGURE 1:**
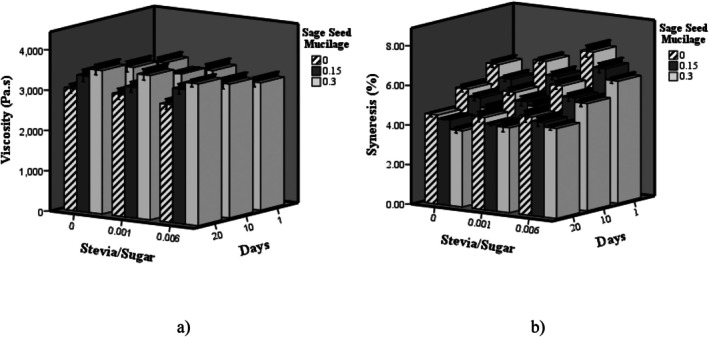
The effect of SSR and sage seed mucilage on the viscosity (a) and syneresis (%) (b) of probiotic dairy dessert during storage.

The data illustrating changes in viscosity and syneresis of probiotic dairy desserts over storage time are presented in Figure 1a,b. Increasing the SSR in the milk dessert samples resulted in a significant reduction in viscosity accompanied by a marked increase in syneresis (*p < 0.05*). Conversely, elevating the concentration of SSM significantly enhanced viscosity and decreased syneresis in the samples (*p < 0.05*). Moreover, the findings indicate that during storage, the viscosity increased significantly while syneresis decreased markedly (*p < 0.05*). In dairy dessert formulations, sugar serves not only as a sweetening agent but also plays a crucial role in interacting with proteins to stabilize the protein gel network. Increasing the SSR reduces the overall sugar content and consequently decreases the dry matter in the formulation, which contributes to a reduction in viscosity. Due to its higher sweetness intensity, stevia is used in much smaller quantities than sugar, and this reduction in total solids further impacts viscosity. Additionally, sugar possesses hygroscopic properties that enable it to absorb and retain moisture within the gel matrix, thereby reducing syneresis. Consequently, increasing the proportion of stevia to sugar compromises the gel network's moisture retention capacity, thereby causing an increase in syneresis (Narayana et al. 2022). The SSM is abundant in polysaccharides and soluble fibers, which possess strong water‐binding and moisture‐retention capabilities within the food matrix. Consequently, increasing the concentration of SSM enhances the water absorption capacity and viscosity of the product, while simultaneously reducing water separation and syneresis (Seo et al. 2018). The results show a significant interaction between stevia and SSM on syneresis and viscosity (*p < 0.05*). SSM helps offset stevia's thinning effect by improving water retention, restoring viscosity, and reducing syneresis. This synergy is important for creating dairy desserts with good texture and stability while reducing sugar. Studies on dairy systems incorporating hydrocolloids have demonstrated that during storage, viscosity increases while syneresis decreases. These effects are attributed to the progressive hydration of the hydrocolloid, enhanced interactions between the hydrocolloid and protein network, and subsequent structural rearrangements within the gel matrix (Basiri et al. 2018). Similarly, Campos et al. (2024) reported increased syneresis in low‐fat yogurt samples by replacing stevia with sugar. In this regard, in the study of basil seed mucilage, a decrease in milk protein gel syneresis has been reported with increasing basil seed mucilage concentration (Song and Kim 2019). In a study investigating the use of jujube mucilage as a stabilizer, an increase in viscosity and a reduction in syneresis were observed in stirred yogurt during the storage time (Yekta and Ansari 2019).

### 3.3 Color Assessment of Probiotic Dairy Dessert

**FIGURE 2:**
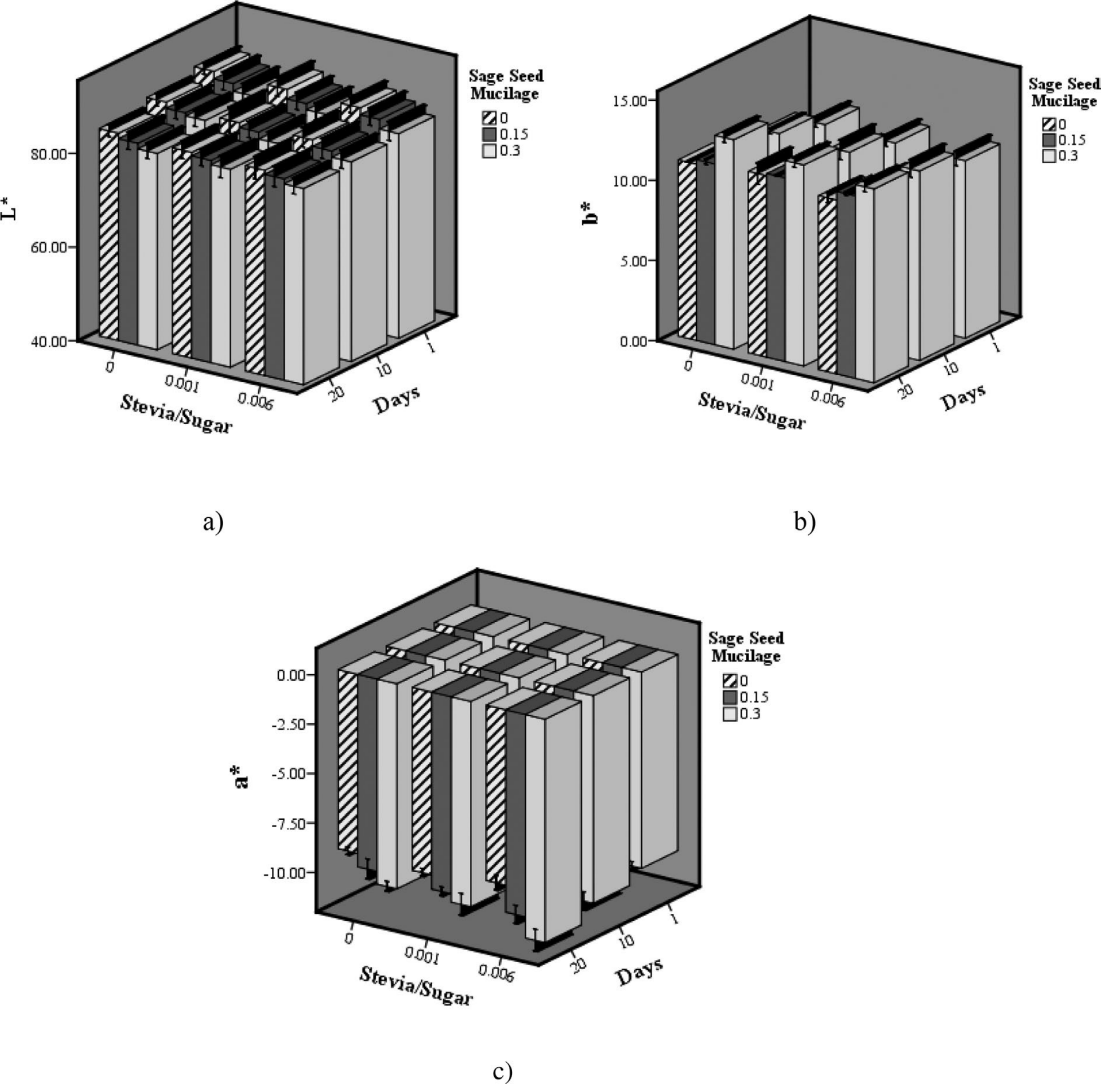
The effect of SSR and sage seed mucilage on color indexes: (a) L*, (b) b*, and (c) a* of probiotic dairy dessert during storage.

Figure 2a,b,c illustrates the changes in the L*, b*, and a* color index of probiotic dairy desserts containing stevia and SSM during the storage period. Increasing the proportion of stevia relative to sugar did not significantly affect the L* (lightness) and b* (yellow‐blue) color indexes (*p > 0.05*); however, a significant decrease was observed in the a* value (red‐green) index (*p < 0.05*). Conversely, elevating the SSM concentration resulted in a significant reduction in both lightness and redness (L* and a*), accompanied by a significant increase in the b* value (*p < 0.05*). Throughout the storage period, the L* and a* indexes exhibited a significant decline, while the b* index increased markedly. Since stevia lacks inherent red pigments, increasing its proportion as a sugar substitute does not enhance the redness of the product (Arslaner et al. 2019). SSM is composed of natural polysaccharides and pigments that contribute a subtle yellowish or off‐white coloration. With increasing concentration, these pigments become more prominent within the dessert matrix, leading to a decrease in overall lightness (lower L*) and a shift in redness (a*) toward neutral or green tones, accompanied by an increase in yellowness (b*). It appears that increasing the concentration of SSM, characterized by its fibrous structure, leads to the formation of a denser gel network, which in turn influences light reflection and reduces the brightness of the dessert samples (Kim et al. 2020). The nonenzymatic browning reaction between reducing sugars and proteins leads to the formation of melanoidin pigments, which contribute to a decrease in brightness (L*) and promote a shift in the color of dessert samples toward brown and yellow hues. Moreover, the oxidation of certain chemical compounds throughout storage generates ketones and aldehydes, which contribute to an increased intensity of yellow coloration. Conversely, the oxidative degradation of red pigments over the storage period diminishes the intensity of the red color in the product (Leal et al. 2022).

### 3.4 Total Phenolic Content and Antioxidant Activity

**FIGURE 3:**
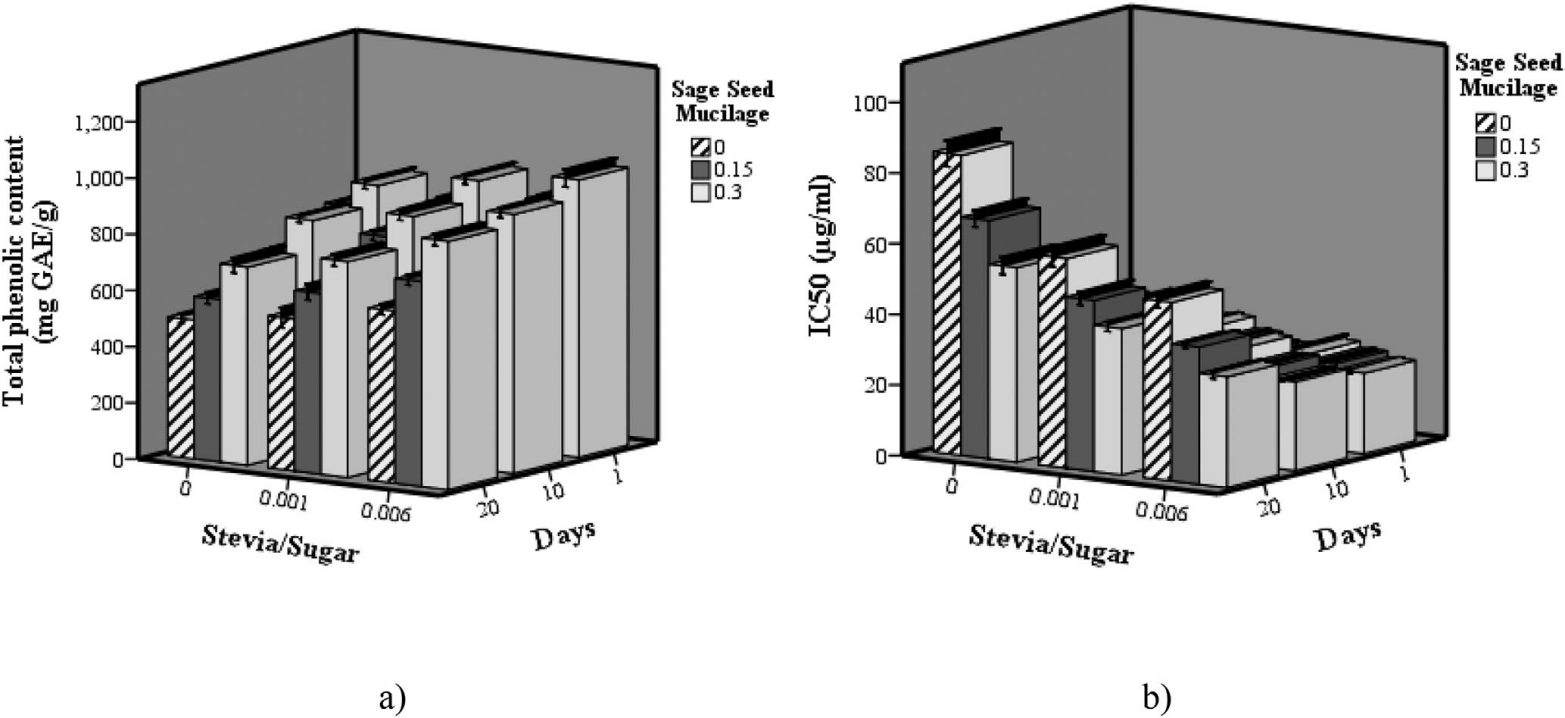
The effect of SSR and sage seed mucilage on total phenolic count (mg GAE/g) (a) and antioxidant activity (IC_50_ (μg/ml)) of probiotic dairy dessert during storage.

The impact of stevia and SSM on the phenolic content (mg GAE/g) and antioxidant activity (IC_50_) of dairy dessert samples during storage is illustrated in Figure 3a,b. Notably, samples with greater antioxidant activity are characterized by lower IC_50_ values, whereas those with diminished antioxidant activity exhibit higher IC_50_ values. Increasing the proportion of stevia relative to sugar in milk dessert samples resulted in a significant enhancement of both phenolic content and antioxidant activity (*p < 0.05*). Similarly, the incorporation of SSM produced a significant and progressive enhancement in these parameters (*p < 0.05*). The results reveal a significant interactive effect of stevia and SSM on phenolic content and antioxidant activity (*p < 0.05*). Despite these initial improvements, both phenolic content and antioxidant activity declined significantly throughout the storage period (*p < 0.05*). These findings align with previous research highlighting the rich phytochemical profile of *Stevia rebaudiana* leaves, which contain various bioactive compounds such as phenolic acids—including chlorogenic and caffeic acids—and flavonoids like quercetin and kaempferol. These compounds are well‐documented for their potent antioxidant properties, primarily through mechanisms involving the donation of hydrogen atoms or electrons to neutralize free radicals. Unlike sucrose, a pure carbohydrate devoid of phenolic compounds, stevia contributes directly to the phenolic content of food products when used as a sugar substitute, thereby enhancing their antioxidant potential (Papaefthimiou et al. 2023). SSM, rich in phenolic compounds, may synergistically enhance the antioxidant activity of dairy desserts when combined with bioactive constituents from stevia (Vosoughi et al. 2018). Supporting evidence from other studies corroborates these results. For example, Akalan et al. (2023) reported a significant increase in phenolic content and antioxidant activity in yogurt fortified with instant stevia powder. Similarly, De Carvalho et al. (2019) found enhanced phenolic content and antioxidant capacity in yogurt samples enriched with freeze‐dried stevia extract. In addition, the phenolic and flavonoid contents, as well as the antioxidant activity of the alcoholic extract of Sage ( *Salvia officinalis* ), have been reported (Vosoughi et al. 2018). The increased phenolic content and antioxidant activity resulting from the addition of stevia and SSM provide extra health benefits for consumers looking for healthier dessert choices. Phenolic compounds and antioxidants help reduce oxidative stress, which is connected to a decreased risk of chronic illnesses like cardiovascular disease, diabetes, and some types of cancer. Consequently, the improved antioxidant capacity of the dairy dessert may support better overall health beyond basic nutritional value (Vosoughi et al. 2018; De Carvalho et al. 2019).

### 3.5 *L. Casei* Viability of Probiotic Dairy Dessert

**FIGURE 4:**
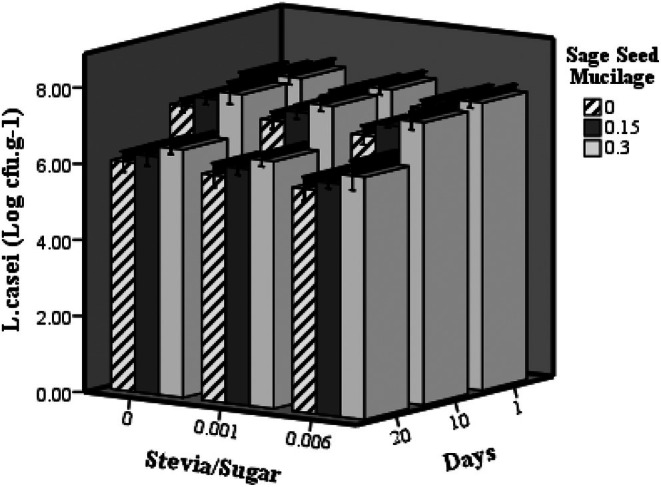
The effect of SSR and sage seed mucilage on *L. casei* (Log cfu.g^−1^) of probiotic dairy dessert during storage.

Figure 4 illustrates the impact of stevia and SSM on the viability of *L. casei* during storage. Increasing the SSR to 0.001 did not significantly affect the survival of the probiotic bacteria; however, further increasing this ratio to 0.006 resulted in a significant decline in *L. casei* viability (*p < 0.05*). Conversely, elevating the concentration of SSM exerted a positive and statistically significant effect on the survival of *L. casei* . Despite these effects, prolonged storage time was associated with a significant reduction in probiotic viability (*p < 0.05*).

Stevia leaf powder appears to contain a diverse array of nutrients that support the growth of probiotic bacteria, whereas stevioside, a concentrated glycoside found in stevia, may inhibit the proliferation of certain probiotic strains (Basharat et al. 2025). Meanwhile, SSM forms viscous, gel‐like matrices that act as physical barriers against environmental stresses such as acidity, oxygen exposure, and temperature fluctuations, thereby aiding in the preservation of probiotic viability. Additionally, the polysaccharides and bioactive compounds present in SSM may function as substrates or prebiotics, enhancing probiotic growth and metabolic activity (Nasiri et al. 2021). Despite these protective effects, probiotic viability tends to decline during storage due to factors including temperature‐induced metabolic stress, oxidative damage from oxygen exposure, moisture‐related challenges, and acidic conditions (Arabshahi and Sedaghati 2022). In this context, Denina et al. (2014) demonstrated that steviol glycosides exert strain‐specific and concentration‐dependent inhibitory effects on the survival of *Lactobacillus reuteri* strains. Similarly, Costa et al. (2019) reported that stevia negatively impacted probiotic survivability under simulated gastrointestinal conditions.

### 3.6 Sensory Assessment of Probiotic Dairy Dessert

**FIGURE 5:**
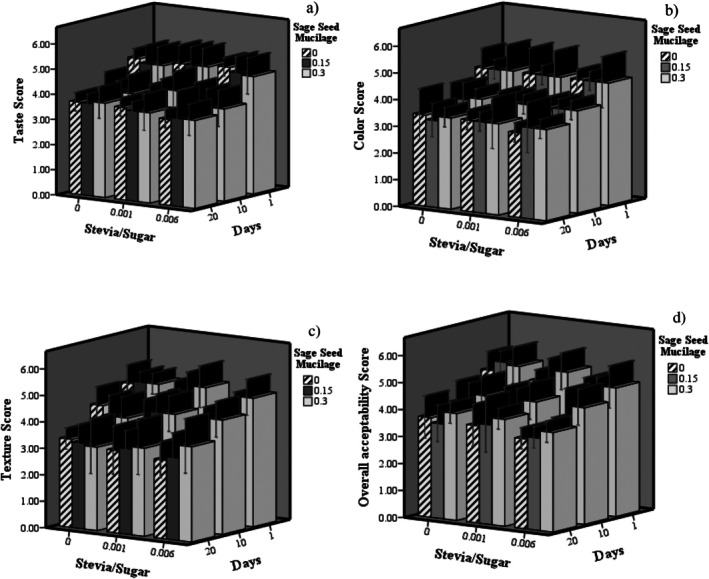
The effect of SSR and sage seed mucilage (SSM) on sensory properties of probiotic dairy dessert during storage: Taste (a), Color (b), Texture (c), and Overall acceptability (d).

Figure 5 presents the comparative sensory evaluation results of the dairy dessert samples, as assessed by panelists during storage, focusing on taste, color, texture, and overall acceptability. Statistical analysis showed that increasing the SSM concentration did not significantly affect taste scores (*p > 0.05*). Although raising the SSR to 0.001% had no significant effect, a further increase to 0.006% resulted in a significant decrease in taste scores (*p < 0.05*). In terms of color, all samples exhibited similar levels of acceptance, with no significant differences observed due to the addition of stevia or SSM, with mean scores of 3.91 ± 0.5. Although a higher SSR resulted in a slight, nonsignificant decrease in texture scores, increasing the SSM percentage produced a significant improvement in texture, with a mean score of 3.89 ± 0.2 (*p < 0.05*). The overall acceptability of the samples remained comparable to the control when the SSR was increased to 0.001%, but a further increase to 0.006% reduced acceptability (*p < 0.05*). Notably, a higher SSM percentage significantly enhanced overall acceptability across all samples (*p < 0.05*), with the highest acceptability seen in samples containing 0.3% SSM (mean score: 3.95 ± 0.2). All sensory attributes—taste, color, texture, and overall acceptability—showed a significant decline during storage (*p < 0.05*). These findings are consistent with previous studies, such as Arabshahi and Sedaghati et al. (2022), who reported improvements in texture and overall acceptability in symbiotic doogh containing *Plantago psyllium* mucilage. Similarly, Kalicka et al. (2017) found that adding stevia up to 0.04% did not negatively affect yogurt flavor, while Bilgiç and Seyrekoğlu (2025) observed a decrease in overall acceptance in fruit yogurts sweetened with stevia during storage. Based on these results, the dairy dessert sample with a sugar‐to‐stevia ratio of 0.001 and 0.3% gum on Day 10 is recommended for production from a sensory perspective, following the control sample.

## 4 Conclusion

This study showed that increasing SSR in dairy desserts reduces sugar content and calories but may negatively affect texture and probiotic viability at higher levels. Adding SSM improves viscosity, reduces syneresis, enhances antioxidant properties, and supports probiotic survival. Sensory analysis identified the formulation with an SSR of 0.001 and 0.3% SSM on Day 10 as optimal, balancing health benefits and consumer acceptability. This formulation is recommended for producing a healthier, functional dairy dessert. The findings provide valuable insights for the development of innovative functional foods aimed at enhancing consumer health. Future research should investigate the long‐term health benefits and explore the synergistic effects of combining these compounds with other bioactive ingredients to maximize their benefit potential.

## Ethics Statement

The authors have nothing to report.

## Conflicts of Interest

The authors declare no conflicts of interest.

## Data Availability

The data that support the findings of this study are available from the corresponding author upon reasonable request.
